# Exploring opportunities to enhance effectiveness of mobile health and nutrition strategy for providing health and nutrition services amongst pastoralists in Somali region, Ethiopia

**DOI:** 10.4102/phcfm.v13i1.2723

**Published:** 2021-04-09

**Authors:** Olusola Oladeji, Bibilola Oladeji, Mohamed Diaaeldin Omer, Abdifatah Elmi Farah, Ida M. Ameda, Rajeev Gera, Abibakar S. Ismail, Mohamed Ayanle, Opiyo Nixon, Hadis M. Diriye

**Affiliations:** 1 UNICEF Somali Region, UNICEF Ethiopia, Jijiga, Ethiopia; 2 Department of Psychiatry, College of Medicine, University of Ibadan, Ibadan, Nigeria; 3 UNICEF ESARO, East and Southern Africa Regional Office, Nairobi, Kenya; 4 UNICEF Ethiopia, Country Office, Addis Ababa, Ethiopia; 5 Somali Regional Health Bureau, Jijiga, Ethiopia

**Keywords:** mobile health team, pastoralist, woreda (district), nutrition, strategy

## Abstract

**Background:**

The health system in Ethiopia’s Somali Region is weak with limited number of health facilities with more than 60% of the population living more than 5 km to the nearest health facilities. The deployment of mobile health and nutrition teams has played critical role in providing essential health and nutrition services.

**Aim:**

This study aimed to assess the effectiveness of the mobile health and nutrition strategy in providing health and nutrition services in the targeted woredas (districts).

**Setting:**

Somali Region of Ethiopia.

**Methods:**

The study was a retrospective chart review of the monthly mobile health and nutrition team and the static health facilities in the 29 woredas between April 2019 and March 2020 and the AccessMod analyses for geographical accessibility to health facilities in the region.

**Results:**

40 (40.4%) out of the 99 woredas in Somali regions have at least 80% of the population living more than 5 km from the nearest health facility out of which 18 (45%) woredas are currently being supported by the mobile health team. The mobile team contributed to increasing access to health services in the targeted woredas with 30.8% of the total children vaccinated for measles and 39% of the total children treated for severe acute malnutrition in the targeted 29 woredas.

**Conclusion:**

With mobile health and nutrition strategy being recognised as a useful strategy to deliver health and nutrition services in the region, there is a need to explore opportunities and innovation to enhance the effectiveness of the implementation.

## Introduction

Ethiopia has made great efforts in recent years to improve maternal, newborn and child health (MNCH) outcomes with the implementation of the country’s health-sector transformation plan and other strategic initiatives that have driven a steady decline in mortality amongst mothers, newborns, infants and children under 5 years.^1^ There are, however, still wide variations in these indicators at the subnational level of Somali Region which is one of Ethiopia’s four Developing Regional States (DRS) and the second largest region in Ethiopia in terms of land mass with population of over 6 million people, predominantly pastoralists (85%), with most people living in hard to reach/remote areas that face developmental inequities.^2^ The region has shown less improvement in key health outcomes compared to the national average; the 2019 Mini Demographic Health Survey (DHS) reports that the region has the lowest percentage of fully vaccinated children, with only 18.2% of children having received all basic vaccinations and 48.8% of children had received no vaccinations at all compared to national average of 43.1% and 19.2%, respectively, whilst the percentage of pregnant women who received at least four antenatal care services (ANC4) and were delivered by skilled birth attendants is 11.1% and 26% compared to the national average of 43% and 49.8%, respectively.^3^

The health system in the region is weak with limited number of health facilities, poor infrastructure, limited number of qualified health workers and maldistribution of the workforce with health facilities in remote areas not well staffed whilst with more than 60% of the population walking for more than 1 h to reach the nearest health facilities regardless of their functionality status.^4^ In addition to being a dominant pastoralist community, the mobility of the community is one of the barriers for the access and utilisation of health services in this pastoralist community. However, in order to improve access to equitable health and nutrition services, United Nation Children Fund (UNICEF) has been supporting the Regional Health Bureau (RHB) in the last 14 years with the deployment of mobile health and nutrition teams. The teams have played a critical role in providing essential health and nutrition services and emergency response to these vulnerable communities, especially the pastoralists residing in areas hard to reach or living in communities without functional health facilities.^5^

This study aims to assess the effectiveness of the mobile health and nutrition strategy in improving access to and utilising health and nutrition services in the 29 targeted woredas (districts) and explore opportunities to enhance its implementation for better results and impact.

### Research methods and design

The study was a retrospective chart review of the monthly mobile health and nutrition database and the monthly service utilisation reports of the static health facilities in the 29 woredas accessed through the District Health Information System (DHIS) database and the preliminary results of the AccessMod analyses^6^ for geographical accessibility to health facilities in the region.

Healthcare accessibility modelling using AccessMod was funded by the UNICEF East and Southern Africa Regional Office to support decision-making on health service expansion in order to reach the largest number of people.

AccessMod is a free and open-source software tool developed to model the physical accessibility of existing health facilities in the target population. To model accessibility to health facilities, the study accounted for topography, road networks, constraints to movement (e.g. rivers and lakes), population distribution of the target population and health facility locations to determine population of those who do not have WHO-defined healthcare access (> 5 km). This accessibility modelling in AccessMod was deployed as an initial step to identify communities with poor healthcare access and where health service expansion through new health facilities or mobile outreach will reach the largest number of people.

The review focused on key health and nutrition services provided through the mobile health and nutrition teams and the static health facilities in the 29 woredas over a period of 1 year (between April 2019 and March 2020). The healthcare accessibility modelling was conducted between December 2019 and March 2020.

### Study population and setting

Since 2004, UNICEF has been supporting the Somali Regional Bureaus with mobile health and nutrition teams, currently deployed to 29 woredas across the 11 zones in the region.

This study focused on assessing the effectiveness of the mobile health and nutrition strategy in improving access to and utilisation of health and nutrition services in the targeted 29 woredas and explored opportunities to enhance its implementation for better impact.

During the reporting period, the 29 mobile health and nutrition teams worked 5 days in a week in different locations in the 29 woredas based on the monthly schedule. Each mobile team is made up of three nurse/midwives, two health extension workers and 1 social mobiliser. The mobile health and nutrition service package incorporates the fundamentals of integrated management of new-born and childhood illness (IMNCI), immunisation, maternal health support (focused Antenatal Care [ANC], post-natal care [PNC] and delivery ), management of severe and moderate acute malnutrition through outpatient therapeutic feeding programme (OTP) and targeted supplementary feeding program (TSFP), promotion of personal sanitation/environmental hygiene and prevention of gender-based violence.

### Data collection and analysis

The data were extracted from the records on service utilisation between April 2019 and March 2020 for the mobile health and nutrition teams and the health facilities in the 29 woredas in the mobile health and nutrition team (MHNT) database and the Regional DHIS database, respectively. The extracted data were entered into a Microsoft Excel spreadsheet and analysed using SPSS version 21. Univariate analysis was done by generating frequencies and proportions and variables presented in graph form.

The healthcare accessibility was modelled by the use of AccessMod, a free and open-source software tool developed to model the physical accessibility of health facilities and presented as graph. The primary outcome is the contribution of the mobile health and nutrition strategy to the service utilisation in the targeted woredas and the secondary outcome is to explore opportunities and innovation to improve the effectiveness of the strategy.

### Ethical considerations

Ethical approval to conduct the study was obtained from the Somali Regional Health Bureau Ethical Committee – SRHB-118-9667.

### Results

Table 1 shows the details of the Mobile Health and Nutrition Team Operation areas highlighting the zones, woredas, the number of health facilities and the number of people in each woreda who live within 1 h walkable distance to the nearest health facilities regardless of their functionality status. The mobile health and nutrition team is implemented in 29 woredas in 11 zones of the region, with an estimated population of 1 706 769. There are also 46 health centres and 236 health posts in these woredas providing essential health and nutrition services. The percentage of the population living within 1-h walkable distance (5 km) from the nearest health facilities ranged from 1.2% in Gablalu woreda to 62.0% in Kalafo woreda with mean of 19.9 ± 6.1.

**TABLE 1:** Profile of mobile health and nutrition team operational areas in Somali region.

S/N	Zone	Woreda	Health post	Health centre	Population	% population within 1 h to the nearest health facility
1	Erer	Yaxob	2	0	19 022	2.0
2		Kubbi	4	1	46 290	11.0
3		Maymuluko	4	1	16 845	4.0
4		Legahida	2	1	24 157	10.0
5		Selahad	5	1	32 844	11.0
6	Doolo	Galxamur	6	1	36 028	14.0
7		Warder	10	2	36 028	12.0
8	Jarrar	Gashamo	12	3	103 572	16.0
9	Korahey	Bodalay	2	0	20 190	25.0
10		Goglo	4	1	21 528	7.0
11		Higloleey	4	1	19 697	10.5
12		Shilabo	8	3	77 422	30.0
13	Dawa	Moyale	35	3	240 614	34.0
14	Shabelle	Adadle	15	3	111 578	29.0
15		Eleele	4	1	10 989	5.0
16		East-Ime	6	1	77 996	50.0
17		Kelafo	17	3	103 686	62.0
18	Nogob	Segage	3	2	49 362	18.0
19		Elwayne	2	1	32 028	4.0
20		Horshagax	1	1	16 416	9.0
21	Fafan	Babile	10	2	103 710	25.0
22		Tulliguled	8	3	66 847	60.0
23		Goljano	5	1	57 567	15.0
24	Afder	Charati	31	2	122 989	22.5
25		West Ime	11	2	64 861	45.0
26	Liban	G/Damole	7	2	28 805	2.5
27		Dhekasuftu	4	1	60 799	27.5
28	Sitti	Gablaalu	2	0	17 840	1.2
29		Afdem	12	3	87 059	16.0

**Total**			**236**	**46**	**1 706 769**	**-**

S/N, serial number.

**FIGURE 1:**
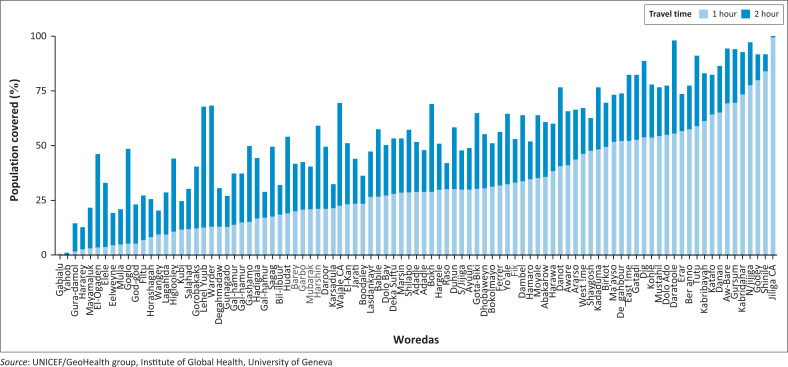
Population coverage by health facilities per Woreda, within 1-h and 2-h travel time, in Somali region, Ethiopia.

Figure 1 shows the results of the geographical accessibility to health facilities in the region highlighting the population coverage per woreda that is within 1 h travel time to the nearest health facilities. It shows that 40 (40.4%) out of the 99 woredas in Somali regions have at least 80% of the population spending more than 1 h travel time (5 km) from the nearest health facility. The proportion of population living away from health facility access varies from as little as 38.0% in Kalafo woreda in Shabele zone to more than 90.0% population in Gabalalu woreda in Sitti Zone with the mean of 80.1% living remotely in the study population.

Of the total of 99 woredas in the region, 18 (45%) woredas were supported by 29 MHNTs that work exclusively amongst pastoralist populations that live more than 1 h walkable distance (5 km) from the nearest health facilities.

Table 2 shows the details of children under 5 years with different health and nutrition services through the 29 mobile health and nutrition teams and the 282 health facilities (236 health posts and 46 health centres) in the 29 project woredas and highlights the contribution of the mobile health and nutrition teams to the overall achievement of the woreda in a year between April 2019 and March 2020.

**TABLE 2:** Children reached with different services through the Mobile health team and Health facilities between March 2019 and April 2020.

Variables	Children fully vaccinated			Children who received Penta1 vaccine			Children who received Penta3 vaccine			Children who received first dose Measles vaccine		
	MHNT	HF	Total	MHNT	HF	Total	MHNT	HF	Total	MHNT	HT	Total
Number reached	5801	25 751	31 552	21 532	46 996	68 528	11 817	38 088	49 905	14 904	33 410	48 314
Mean	156.8	888	-	384.6	1620.55	-	277.38	1313.4	-	241.68	1152	-
Standard error of mean (SEM)	34.46	165.3	-	45.11	239	-	37.5	209	-	97.18	199.5	-

*Source:* Somali Regional Health Bureau Demographic Health Information System and Mobile Health Team database

MHNT, Mobile Health and Nutrition Teams; HF, health facilities.

The mobile health team vaccinated 21 532 children with a mean of 384.5 per mobile team with the 1st dose of pentavalent vaccine (Penta 1) compared with 46 996 children vaccinated at health facilities with a mean of 1620.5 per health facility. It shows that 21 532 (31.42%) of 68 528 children under 1 year in the 29 woredas received the first dose of pentavalent vaccine (Penta 1) through the mobile health teams.

Similarly, through the mobile health team, 5801 children with a mean of 156.8 per mobile team were fully vaccinated compared to 25 751 children fully vaccinated at health facilities with a mean of 888.0 per health facility. It shows that 5801 (18.35%) of the total number of children who were fully vaccinated were approached by the mobile health teams.

However, in terms of continuity of services using the dropout rate between children who received the first dose of pentavalent vaccine and those who received the third dose of pentavalent vaccine, it shows that through the mobile health team, 21 532 children received the first dose of pentavalent vaccine compared to 11 817 children who received the third dose of pentavalent vaccines with a difference of 9715 children who dropped out giving a dropout rate of 45.1%. Amongst the 46 996 children who received the first dose of pentavalent vaccine through the health facilities, 38 088 received the third dose with 8905 children who dropped out giving a dropout rate of 18.9%.

Table 3 shows other services provided during the reporting period. A total of 6732 children of 6 months to 5 years with a mean of 147.0 per mobile team with several acute malnutrition were treated through the OTP by the mobile health and nutrition team compared to 10 499 children with severe acute malnutrition treated at health facilities with a mean of 362.0 per health facility. This shows that 6732 (39.1%) of all the 17 231 children of 6 months to 5 years with severe acute malnutrition were treated through the OTP in the 29 woredas by the mobile health and nutrition team.

**TABLE 3:** Children reached with different services through the mobile health team and health facilities between March 2019 and April 2020.

Variables	Outpatient Under 5 consultation			Children treated for Severe Acute Malnutrition (OTP)			
	MHNT	MHNT	HF	Total	MHNT	HF	Total
Number reached	431	134 796	51 638	186 434	6732	10 499	17 231
Mean	11.34	300.64	1781	-	147	362.03	-
SEM	6.99	61.27	319	-	19.9	123.94	-

*Source:* Somali Regional Health Bureau Demographic Health Information System and Mobile Health Team database

MHNT, Mobile Health and Nutrition Teams; HF, health facilities; OTP, outpatient therapeutic feeding programme; SEM, Standard error of mean.

A total of 134 796 children under 5 years with a mean of 300.6 per mobile team were treated for common ailments during the outpatient consultations by the mobile health team compared to 51 638 children with common ailment treated at health facilities with a mean of 1781.0 per health facility.

This shows that 134 796 (72.3%) children under 5 years were treated for common ailment during outpatient services provided by the mobile health team.

## Discussion

The delivery of health services through mobile clinics has been recognised as the most effective approach for service delivery for the pastoralists, conflict affected areas and hard to reach areas as reported in studies amongst pastoralists in Chad, the Sahel and conflict affected and remote area in Nigeria and Afghanistan.^7,8,9,10,11^ These studies reported improved access and utilisation of primary healthcare for women and children including immunisation services. The findings in this study with the contribution of the mobile health and nutrition teams to the overall health and nutrition service utilisation, especially for children, also reaffirmed the importance of this strategy to improving access to the vulnerable communities hard to reach and pastoralist communities. This shows a significant contribution of the mobile health and nutrition strategy to access and utilisation of services by 29 teams compared to 282 health facilities (236 health posts and 46 health centres). However, using the number of children who dropped out from the immunisation services using the pentavalent vaccine, it shows that in both the health facilities and the mobile health team, there is significant dropout rate when compared to the acceptable dropout rate of less than 10% with the dropout rate in the mobile health team being very high. This is because of the mobility pattern and lifestyle of the communities and therefore, there is a need to develop appropriate strategy to reduce the dropout rate.

However, unlike most mobile health teams which provide only healthcare services, the mobile health team in this study provided in addition nutrition services for children with moderate acute malnutrition and severe acute malnutrition which helped in reaching children with acute malnutrition in a region with the highest prevalence of wastes in the country.^3^ In addition, different service delivery modalities are adopted for the mobile health team in different settings from periodic team movements from one pastoralist zones every 2 weeks as documented by a study in Kenya or combination of mobile and fixed services in Niger, or fewer days like for 1–2 days every 2 months or quarterly visits to each different sites,^10^ however in this study, the mobile health teams are assigned every week day in different locations/settlements within a woreda.^10,11,12,13^

One of the major challenges to providing services to the pastoralists is the seasonal movement that affects their utilisation of healthcare services, as they usually move further away from existing services delivery points. The regular movement of nomads acts as a barrier for women and children to access maternal and child healthcare services needed to ensure universal health coverage and improve on maternal and child health and nutrition outcomes. The situation is even worse, as most of the mobile health teams are usually not available to accompany the groups as they relocate during the seasonal movement from one location to other seeking for pasture for their animals.^14^ This is likely a major issue with the implementation modality used in the mobile health team strategy in this study where the teams are assigned 3–6 months in specific locations in the woredas or some more than a year.^5^ In such setting, during the seasonal movement by the pastoralist, many of them move away from where the services are available to other locations and may not be aware of the location of the MHNT in other areas they move to and thus unable to access services during this period until they return to their bases at the end of the season. In order for the mobile health teams to be more effective, there is a need for flexibility and review of the implementation strategy that ensure that the pastoralists continue to access basic and essential health and nutrition services during their seasonal movements.

The seasonal movements of the pastoralists require regular information and demographic surveillance about them for effective planning to ensure access to services. There is a need to explore the opportunities for the possibility of tracking the movement of the pastoralists during their seasonal movements, so they could be reached with targeted health and nutrition services to reduce their use of unorthodox medical care with associated morbidity and mortality.

Studies in Ethiopia, Senegal and amongst Fulani in west Africa have shown that in most of the pastoral communities, the seasonal movements are well-planned and targeted between wet and dry season grazing areas and they usually have their mobility plan which is well organised and sometimes under the supervision of traditional institutions.^4,15,16^

In addition, the pastoralists have their own well-established communication systems for sharing information on climatic conditions and ecology, as well as economic, social and political issues including the use of mobile telephones which is increasingly being used by pastoralists to access information relevant to their livestock all-round the year even during the seasonal movements.^17^ Studies in Tanzania, Nigeria and Chad have documented the use of mobile phone to link pastoralists with information to improve their productivity and value chains.^18,19,20^ The study in Chad used the mobile phone to conduct demographic surveillance of humans and animals amongst the pastoralists and showed the feasibility of mobile surveillance of mobile pastoralists camps and provided usable reliable, nearly real-time information on human and livestock population structures, pregnancy outcomes and herd dynamics, as well as mobility patterns which provides an opportunity for specific measures and maximises the spatio-temporal accessibility of the camps for social service providers, aiming to increase coverage of specific interventions.^20^

With the improvement in the mobile phone connectivity coverage and ownership and use of mobile phone in Ethiopia with 90% coverage and 71% ownership and use of mobile phone in Somali region,^21,22^ a communication system can be established/explored between the leadership of the pastoralists and the mobile health team operation centres to identify periodically the locations of the pastoralists, especially during the seasonal movements. The real-time knowledge on camp locations and populations would facilitate health interventions such as vaccination, treatment of common ailments, acute malnutrition, Vitamin A supplementation, deworming tablet for children and iron and folate for pregnant and lactating mothers and contraceptives and hygiene promotion or sensitisation and information campaigns on maternal and child health. Additionally, outbreaks of human and animal diseases, like cholera, measles or any zoonotic diseases, could be monitored closely, enabling control measures to be quickly implemented.

The geographical health accessibility report in this study shows that 40 out of the 99 woredas in Somali regions have at least 80% of the population spending more than 1 h of travel time (5 km) for a health facility out of which 18 woredas are currently being supported by the mobile health teams. This shows that based on the WHO recommendation using 5 km walkable distance to the nearest health facility as measure of access to healthcare services, a large part of the Somali communities lacks access to equitable healthcare services.^23^ This study demonstrates that the use of relevant evidence-based information like the geographical accessibility modelling analysis in helping to identify the population mostly in need of services should be prioritised in order to have maximum impact by reaching a large population with limited or no accessibility to health facilities.

Geographic assessment to identify the optimal sites for new health facilities or deploying a mobile health team is becoming relevant and increasingly being used to achieve maximum population level increase in accessibility to care. This tool can also be used as a policy guide in ensuring equitable access to health services.^24,25,26,27^ The study in a rural community in South Africa reported that the use of geographic mapping for the sites the mobile health teams were deployed helped in improving access and coverage.^26^ However, the study in India that evaluated the deployment of mobile health team in an underprivileged communities reported poor performance of the mobile health teams in terms of coverage and beneficiaries and even more than 80% of the served population used the services but 90% of the users travelled less than 1 km to avail the service of the mobile health teams, and it is recommended that the need for geographical assessment to identify sites will maximise the coverage and help improve the performance.^27^

Bearing in mind the high operation cost of mobile health teams, although not addressed in this study, it is highly imperative that locations with high population and limited access are prioritised for the deployment of the mobile health team for effective and efficient use of resources and ultimately equitable access to service delivery.

### Limitations

This study is based on clinical records and thus subject to the limitations associated with studies utilising routine data affected by the quality of the data used. The study did not analyse the operational costs of the mobile health team strategy and the health facilities to determine the cost-effectiveness of the strategy for the delivery of essential health services. In addition, the study did not compare health outcomes between pastoral populations with and those without access to MHNTs.

## Recommendations

With mobile health and nutrition strategy being recognised as a useful strategy/platform to deliver health and nutrition services in the region with many donors and NGOs supporting this, there is a need to tweak the operations of the mobile health team to be more flexible, innovative and responsive to the need of the pastoralists and targeting large population with limited or no access to health services. This will include the use of phone for real-time knowledge on movement of the pastoralists and camp locations, so they can be linked to the nearest mobile health team or can be provided with the needed services. In addition, the strategy should be adopted as an alternative primary healthcare service delivery strategy in each woreda (district) in the region to enhance access to primary healthcare service and included in the government budget and managed by each woreda (district) instead of the current arrangement if largely funded by partners and centrally managed from the regional level so as to ensure sustainability and reduction in administrative and operational cost. This will ensure equity and attainment of universal health coverage in the region which ultimately may help improve on abysmally poor health and nutrition indicators and contribute to the attainment of the SDG targets by the country. There is a need for further studies that will compare health outcomes between remote pastoral populations that do and do not have access to MHNTs.

## Conclusion

The mobile health and nutrition strategy has been a useful mechanism to deliver health and nutrition services to the pastoralist population with no or limited access to health services in the targeted woredas in the region in the last 16 years. The strategy should, however, be adopted and institutionalised as an alternative primary healthcare service delivery strategy in all the woredas (districts) in the region to enhance access to primary healthcare service for pastoralist communities and those having difficulties to reach areas with limited and no access to functional health facilities instead of the current arrangement where it is managed as a parallel programme with support mainly by partners. There is a need to explore opportunities and innovation to enhance the effectiveness of the implementation of the strategy.
